# Bilan d’une cure chirurgicale d’aspergillome pulmonaire secondaire a une lesion sequellaire de tuberculose au CHU/JRA

**DOI:** 10.11604/pamj.2013.14.83.2413

**Published:** 2013-03-05

**Authors:** Narindra Njarasoa Mihaja Razafimanjato, Andriamihaja Jean Claude Rakotoarisoa, Manjakaniaina Ravoatrarilandy, Auberlin Felantsoa Rakototiana, Francis Allen Hunald, Luc Hervé Samison, Agnès Marie Lydia Ravalisoa, Danitrala Jean Louis Rakotovao

**Affiliations:** 1 Service de Chirurgie Thoracique CHU/JRA BP, 4150 CP, 101 Antananarivo, Madagascar; 2 Service de Chirurgie viscérale CHU/JRA BP, 4150 CP, 101 Antananarivo, Madagascar; 3 Service de Chirurgie Cardio-vasculaire CHU/JRA BP, 4150 CP, 101 Antananarivo, Madagascar

**Keywords:** Aspergillome pulmonaire, tuberculose, chirurgie, pulmonary aspergilloma, tuberculosis, surgery

## Abstract

La prise en charge d’un aspergillome pulmonaire, dans le cas des lésions limitées accessibles, est une des activités courantes en chirurgie thoracique dans les pays endémique à la tuberculose comme Madagascar. Sur une période allant de janvier 2005 en mars 2010, 15 patients, ancien tuberculeux, atteints d’une aspergillome pulmonaire sont traités par une résection segmentaire ou une lobectomie. La circonstance de découverte repose sur la clinique par des tableaux très polymorphes. L’imagerie garde une place importante. L’examen histologique des pièces d’exérèse chirurgicale confirme le diagnostic. Tous les patients ont été opérés de manière élective. Le résultat a été pour l’ensemble des patients jugés satisfaisant. Ces patients sont suivis pendant 1 à 39 mois. L’étude des résultats à distance est encore en cours et est fondamentale si le traitement chirurgical a un effet bénéfique sur la survie et la qualité de vie des malades. Le but de ce travail a été, à partir de la revue de la littérature et de notre petite expérience, de définir quels éléments pertinents mis en exergue sur le sujet.

## Introduction

Les formes cliniques pathologiques secondaires à l’exposition à Aspergillus sont multiples [1]. Mais seules les formes saprophytes font l’objet de notre intérêt dans cette étude. L’aspergillome classique est une infection saprophyte qui résulte de la colonisation de spores d’aspergillus fumigatus organisés en feutrage mycélien dense d’une cavité pulmonaire préexistante en particulier la caverne tuberculeuse détergée et dépourvue d’un système de défense locale [2, 3]. La chirurgie reste le traitement de référence de cette forme clinique en dépit des difficultés opératoires [3]. Elle permet à la fois de stériliser le foyer de l’aspergillus, par une résection complète de la truffe aspergillaire avec sa cavité et d’éviter les récidives [4]. L’évolution rapidement fatale et les difficultés thérapeutiques rencontrées nous semblent intéressantes à prendre en considération en cas d’aspergillome pulmonaire dans notre centre où les moyens de prise en charge sont encore insuffisants. Nous avons mené cette étude afin d’évaluer le profil actuel de cette affection, ainsi que les résultats de sa prise en charge et nous proposons, à partir de l’expérience de notre service, un arbre décisionnel dans la prise en charge de cette pathologie en milieu précaire comme le nôtre.

## Méthodes

Il s’agit d’une étude rétrospective descriptive allant de janvier 2005 à décembre 2010. Nous avons pris en charge 15 patients porteurs d’une aspergillome bronchopulmonaire secondaire. Il s’agissait de 8 femmes et 7 hommes d’une moyenne d’âge de 33 ans (26 à 58 ans). Parmi notre population d’étude, 14 patients était tous des patients anciens tuberculeux traités et guéris avec négativité des examens des crachats BAAR. Un patient a présenté un cancer broncho-pulmonaire greffé de tuberculose et d’aspergillome pulmonaire.

## Résultats

Le délai d’apparition de l’aspergillome, chez nos malades, est très variable, allant de 10 mois à 12 ans (6, 86 ans +/- 2,87) après la guérison de la tuberculose. La lésion est de forme localisée et simple dans 11 cas au niveau de l’apex droit, 3 cas siégeant au niveau de l’apex pulmonaire gauche. Nous avons répertorié un cas de forme évoluée et complexe. L’hémoptysie est le maître symptôme chez 11 patients (85% des cas). Les autres manifestations cliniques sont la toux, l’expectoration, la dyspnée et l’altération de l’état général (3 patients nécessitent une mesure de réanimation avant l’intervention chirurgicale). Sur le plan radiologique, on objective 9 images (61.53%) en grelot, 4 opacités pseudo-tumorales (23.07%) avec opacités plus ou moins arrondies sans croissant gazeux, 1cas (7.46%) d’image hydroaérique et enfin 1 cas (7.46%) d’image de lésion séquellaire. La fibroscopie bronchique a permis de faire des prélèvements, des aspirations bronchiques pour le diagnostic mycologique avec large prédominance du type fumigatus (12 cas) et 1 cas d’aspergillus flavus. La sérologie était positive dans 10 cas (66.66%). Dans notre étude, le temps pré opératoire est une étape particulièrement importante de la prise en charge des patients adressés pour le traitement chirurgical d’une aspergillome bronchopulmonaire. Cette prise en charge, pour être rationnelle doit prendre en compte plusieurs données en particulier la forme radiologique de l’aspergillome, l’état général et l’état fonctionnel cardio-respiratoire du patient. Dans notre série, 12 cas ont subi d’une intervention chirurgicale d’emblée, les 3 cas ont reçu un traitement médical avant l’intervention définitive de l’aspergillome (dont un patient a bénéficié d’un traitement local en percutané suivi d’une thoracoplastie et deux patients avaient bénéficié d’une chirurgie après 3 mois de réévaluation). Notons que la forme localisée de la maladie constitue un élément très important pour l’évolution et le pronostic de la pathologie. La truffe aspergillaire a été réséquée après une thoracotomie postérolatérale chez 8 patients. Chez 4 patients, l’intervention a été modifiée en une thoracotomie latérale sans section musculaire. Le geste chirurgical dépendait du siège et du volume occupé par la lésion. Dans 11 cas le traitement a été uniforme et a consisté en une lobectomie supérieure droite ou gauche. Une résection du segment apical droit était réalisée dans 3 cas. La libération du poumon s’avérait difficile et laborieuse chez 3 malades.

Tous les patients ont été cliniquement et radiologiquement guéris de leur aspergillome pulmonaire. Les hémoptysies disparaissaient après la chirurgie et aucun malade ne présentait pas de récidive. La morbidité a été dominée par la survenue d’un pyothorax et d’une persistance caverneuse, chez deux patients, ayant évolué favorablement après une pleurostomie et une spéléotomie. Il n’y avait pas de décès en postopératoire. La durée de l’hospitalisation était très variable de 6 jours à 2 mois. L’examen anatomopathologique de la pièce opératoire était systématique dans notre service. Cet examen permettait de confirmer le diagnostic de l’aspergillome pulmonaire et permet de mettre en évidence toutes les formes d’associations lésionnelles possibles dont une forme associée à un cancer broncho-pulmonaire.

Tous les malades ont été suivis avec un recul moyen de 11.5 mois et des extrêmes de 1 à 39 mois. Pendant ce suivi, 2 malades ont été perdus de vue successivement à 8 mois et à un an. Les autres patients ne présentaient aucune séquelle en relation avec leurs lésions pulmonaires. Il n’y avait pas une altération de la fonction respiratoire et la compliance pulmonaire est satisfaisante.

## Discussion

Cette étude nous a permis de colliger un nombre important d’aspergillomes qui sont apparues dans la majorité sur des séquelles de tuberculose. Elle nous a permis de montrer l’intérêt de la prise en charge chirurgicale malgré quelques complications postopératoire non mortelles. Cependant, elle comportait quelques limites car était rétrospective et certains dossiers étaient incomplets. L’aspergillome broncho-pulmonaire est une pneumo-mycose opportuniste résultant le plus souvent de la colonisation de cavités parenchymateuses préexistantes détergées par un champignon du genre aspergillus [5]. Cependant, l’existence d’une cavité n’est pas obligatoire car l’aspergillus peut, grâce à des sécrétions enzymatiques, entraîner une lyse du parenchyme en particulier fragilisé par une radiothérapie antérieure [6]. Parfois même, l’aspergillome survenait sans antécédent pathologique antérieur particulier [5], ce que nous n’avons pas pu observer au cours de notre expérience. Le sex ratio est proche de celui de Kim et al en Corée qui avaient recruté dans son étude autant d’homme et de femme [7]. En revanche, la plupart des auteurs dans la littérature avaient rapporté une prédominance masculine dans cette pathologie avec un âge moyen similaire au nôtre [5]. La tuberculose pulmonaire était l’affection sous-jacente la plus fréquemment observée dans la plupart des séries [5]. Parfois d’autres étiologies peuvent être notées comme une sarcoïdose avancée, une bulle d’emphysème, un kyste hydatique détergé, des bronchectasies, voire même un abcès à pyogènes, un cancer nécrosé ou une pneumopathie excavée [6]. Dans notre étude, nous avons observé un cas d’aspergillome et cancer broncho-pulmonaire associés après tuberculose pulmonaire [8]. Le délai moyen d’apparition des symptômes par rapport à cette tuberculose était de neuf ans environ dans notre étude. Il apparaissait plus court que ceux retrouvés en Afrique noir et en Maghreb, respectivement 9 ans et 14 ans [5, 9]. Les signes cliniques des aspergillomes pulmonaires sont très polymorphes. L′hémoptysie récidivante sous forme de crachats hémoptoïques est l′un des symptômes le plus fréquent. Parfois, même mortelle suite à une hémorragie cataclysmique [3.4]. Dans notre série on a observé un cas d’hémoptysie de ce genre. Sur le plan biologique, la mise en évidence de l’aspergillus en particulier l’aspergillus fumigatus n’a de valeur que dans des prélèvements particuliers (produits de brossage protégé, lavage broncho-alvéolaire, expectorations protégées) [6]. Le sérodiagnostic est un examen clef. La présence d’au moins 4 arcs de précipitation confirme le diagnostic [3]. Les lésions radiologiques siégeaient préférentiellement au niveau des lobes supérieurs et/ou gauche et l’image caractéristique en grelot était mise en évidence chez 20 patients (57.14% des cas).

L’aspect radiologique est très variable allant de l’image très évocatrice en «;grelot »; (opacité endocavitaire surmontée d’une clarté) qui se voit dans plus de 60% des cas [3] jusqu’à l’aspect de poumon détruit aspergillaire [6]. Aucun signe radiographique ne permet de prédire la gravité de l’hémoptysie [4]. La tomodensitométrie (TDM), grâce à sa haute résolution spatiale reste l’examen de choix pour l’analyse sémiologique et topographique des lésions et permet de prévoir les difficultés de la résection sur l’importance de la pachypleurite, de l’épaississement de la paroi de la cavité, des adhérences avec les organes médiastinaux et la mise en évidence des vaisseaux néoformés en territoire extrapleural [2, 3]. La TDM paraît surtout intéressante pour les techniques de drainage percutané en étudiant l’environnement vasculaire, pariétal et parenchymateux de la cavité à injecter an d’éviter une blessure vasculaire [6] La fibroscopie bronchique garde une place importante dans la recherche des cancers proximaux siégeant sur les grosses bronches. L’analyse cytologique des crachats et des liquides d’aspiration bronchique permet d’isoler les Aspergillus et de rechercher des cellules malignes [8]. L’examen anatomopathologique permettait de poser le diagnostic de certitude chez la grande majorité des patients et permet de redresser un diagnostic préopératoire parfois erroné [5]. Les associations plus couramment retrouvées étaient un aspergillome et un cancer bronchopulmonaire [5, 8]. Dans la plupart des cas, cette association morbide constitue une surprise anatomo-pathologique après l’examen histologique des pièces opératoires, telle pour notre cas [8]. En dé’nitive le diagnostic d’aspergillome pulmonaire repose sur un faisceau d’arguments cliniques, radiologiques et biologiques. Une hémoptysie survenant dans un contexte d’une image radiologique suggestive doit faire évoquer le diagnostic qui sera étayé par le dosage des anticorps sériques et confirmé par l’examen histologique de la pièce opératoire.

**Figure 1:**
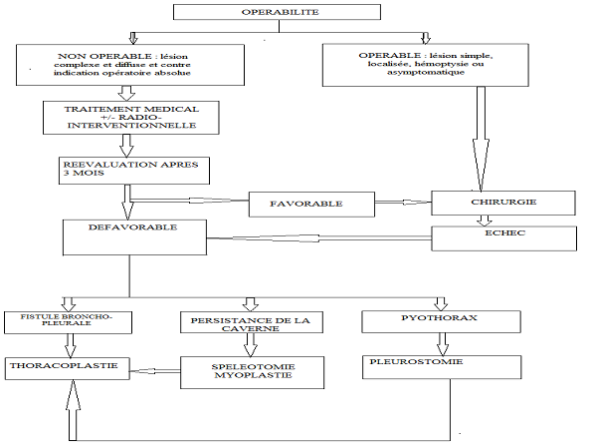
Arbre décisionnel de prise en charge des aspergillomes pulmonaires au CHU/JRA à Madagascar

Cette affection nécessite alors un traitement approprié. Plusieurs moyens ont été proposés et historiquement, la première résection chirurgicale avec succès est réalisée par Gerstyl, Wideman et Newmann en 1948. [10] La prise en charge des aspergillomes pulmonaires réclame une attitude thérapeutique stricte et un arbre décisionnel bien adapté à chaque situation et selon le plateau technique disponible comme nous avons proposé dans cette étude (Figure 1). L’attitude thérapeutique devant l’aspergillome pulmonaire est généralement chirurgicale. Le traitement percutané radiologique a des indications particulières mais l’abstention thérapeutique n’est pas admise même si quelques cas de disparition spontanée par résorption de la truffe aspergillaire ont été rapportés dans la littérature [6]. Nous pensons avec d’autres que l’exérèse chirurgicale est le geste qui semble la plus logique face à des lésions simples et localisées et chez les patients qui ne présentent aucune contre indication de la résection chirurgicale car l’aspergillus peut déclencher des hémoptysies fatales imprévisibles [3, 11]. Le traitement chirurgical permet d’éviter par ailleurs l’évolution vers les formes complexes et diffuses ou invasives chez les patients à profil immunologique précaire [12]. Et l’augmentation progressive de la taille de la truffe aspergillaire qui accentue la néo-vascularisation et les adhérences pariétales et scissurales, ce qui rend l’intervention beaucoup plus difficile et très hémorragique [6]. Cette ablation chirurgicale a pour but d’éradiquer les foyers des mycétomes. La voie d’abord chirurgicale était la thoracotomie postérolatérale pour tous les patients le plus souvent au quatrième espace intercostal. Ceci permet de bien individualiser la truffe aspergillaire dès l’ouverture de la cavité pleurale. Nous avons réalisé que la même dynamique était notée chez beaucoup d’autres auteurs [5]. Les résections chirurgicales pratiquées dans ce contexte ont eu des résultats généralement satisfaisants avec un taux de mortalité opératoire d′environ 7 à 8% [11, 13]. Pour les patients inopérables d’emblée, nous préconisons dans notre centre un traitement médical couplé à un traitement local intracavitaire d’antifongique en percutanée pour stériliser le foyer des mycétomes. Néanmoins, les résultats du traitement par injection intracavitaire de médicaments antifongiques ne sont cependant pas encore assez documentés pour que ce traitement médical puisse notamment se substituer au traitement chirurgical [11]. L’échec de ce traitement médical est sanctionné d’une simple spéléotomie «; truffectomie »;, suivie d’un affaissement de la cavité résiduelle par thoracoplastie ou d’un comblement de cette cavité par myoplastie ou d’une pleurostomie en cas de suppuration pleuro-pulmonaire [6]. Dans les formes symptomatiques et inopérables, d’autres alternatives peuvent être proposées [14, 15]: L’embolisation des artères bronchiques qui n’est pas toujours efficace pour contrôler les hémoptysies massives à cause de l’importance de la circulation collatérale, pariétale, intercostale, transpleurale [6].

Le traitement percutané sous contrôle TDM évitant les inconvénients du drainage percutané, avec utilisation d’une pâte d’amphotéricine B. Le technique du plombage, préconisée par Krakowka, est efficace, et présente peu de complications. Elle est la technique de choix pour les patients précaires ou ceux dont l’imagerie montre des lésions complexes [16]. L’instillation intracavitaire d’iodure de sodium ou de potassium an d’arrêter les hémoptysies chez les patients à haut risque [6].

Les complications postopératoires sont fréquentes avec un taux qui varie entre 15 et 78% des cas [3]. On constate que les lobectomies donnent moins de complications à condition que le parenchyme restant soit de bonne qualité, lui permettant de s’expandre pour combler la cavité résiduelle [3]. Les pneumonectomies ont un taux important de morbidité [3]. L’hémothorax postopératoire se voit dans 7.5 à 75% des cas en raison du caractère hémorragique de ce type d’intervention [3]. La mortalité du traitement chirurgical de l’aspergillome pulmonaire est très variable selon les séries: 4.5% pour Faulkner et al. Et 9% pour Garvey et al, surtout fonction de l’état général du patient et du parenchyme pulmonaire sous-jacent [6].

Au total, le traitement chirurgical des aspergillomes pulmonaires, par exérèse pulmonaire, a un pronostic directement lié au terrain. Cette méthode chirurgicale occupe, jusqu’à maintenant, la première place pour traiter les aspergillomes pulmonaires.

## Conclusion

La prise en charge thérapeutique des aspergillomes broncho-pulmonaires doit être bien codifiée dans les pays où les problèmes thérapeutiques abordés peuvent se poser. Le bénéfice de la prise en charge chirurgicale par exérèse des aspergillomes pulmonaires est évident. Par contre, c’est une chirurgie délicate faisant appel à une stratégie claire impliquant une lésion localisée de l’aspergillome et un état cardio-respiratoire favorable pour une résection pulmonaire. Enfin, ces dossiers doivent être discutés au cas par cas, en comité multidisciplinaire, par analogie aux néoplasies bronchiques.
